# Instant Artificial Bone Preparation From Silicone and Resin: Need of the Hour

**DOI:** 10.7759/cureus.96122

**Published:** 2025-11-05

**Authors:** Jakkula Akhil, Nutan Nalini Bage, Rema Devi

**Affiliations:** 1 Anatomy, Pondicherry Institute of Medical Sciences (PIMS), Puducherry, IND

**Keywords:** artificial bone, general purpose resin, liquid silicone rubber, plastination, silicone mold

## Abstract

Osteology is an important foundation for medical students to understand the framework of the human body. In the recent past, there has been a scarcity of natural resources of human bones, while the number of students and colleges has been drastically increasing. To overcome this challenge, artificial bones came into existence, but they lacked accurate anatomical details and took a few days for the entire process. Therefore, artificial bones that are less time-consuming, cost-effective, and close to the original bone are needed.

The aim of the study is to devise a rapid method of artificial bone preparation. Liquid silicone rubber (LSR-2) was used to prepare silicone molds initially. These molds were used to prepare artificial bones using general-purpose resin (GP resin). The time taken for the entire procedure is less than 90 minutes. The artificial bones were close to natural bones in color, texture, hardness, and weight. Anatomical impressions were well appreciated. These bones could be stored in a routine manner and could be handled safely with bare hands. The present methodology would help in making artificial bone preparation rapidly to cater to a large number of medical students in a short span of time.

## Introduction

Osteology is an important branch of anatomy for the undergraduate basic teaching and learning process. It is essential for specialty and super specialty courses such as orthopedics, surgery, neurosurgery, etc. Lastly, it is needed for training paramedical and allied health sciences.

The practical hands-on learning by students requires a good collection of bones. In the current era, there is a drastic increase in the number of medical colleges and the number of students year by year [1]. At the same time, we are faced with limited resources, but of low quality and high costs. Owing to the increased demand for bones, reduced supply, and exorbitant cost involved, many are obtaining artificial bone models sold commercially [2]. However, such bone models very often lack intricate details of natural bones. The need of the hour to overcome these challenges is to obtain high-quality, cost-effective resources in less time.

Earlier studies were performed using microporous hydroxyapatite, synthetic resin & inorganic fillers, silicone molds & general-purpose resin (GP resin) to prepare artificial bones [3-5]. The existing methodology using silicone molds and GP resin takes nearly two days to obtain the bones [3]. Our current methodology using silicone molds & GP resin would be useful to prepare artificial bones rapidly within 90 minutes, resembling natural bones with finer anatomical details, which is also cost-effective and long-lasting.

## Technical report

Artificial bone making involves creating a mold followed by bone preparation. The entire procedure could be finished within 90 minutes. Materials required can be easily procured at a low cost. Main materials are: Liquid silicone rubber, general purpose (GP) resin, petroleum jelly, etc. The axis cervical vertebra was selected because of its relative scarcity compared to other bones. It was easier to demonstrate mold preparation in two halves due to its smaller size. The procedure was conducted in a well-ventilated area using personal protective equipment (PPE) to avoid inhalation of the resin vapors.

Mold preparation

**Figure 1:**
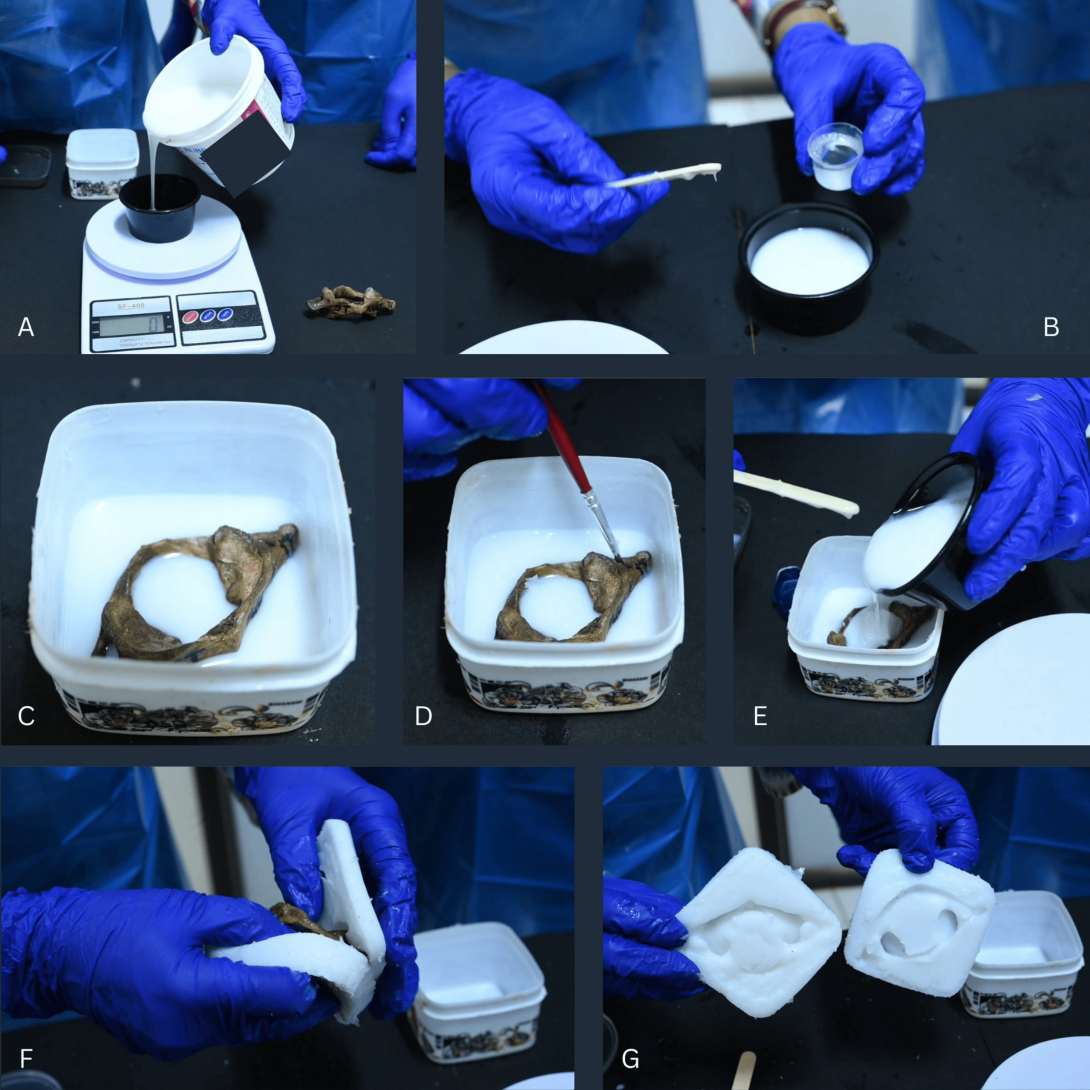
Preparation of Silicone Mold A) Measurement of liquid silicone rubber (LSR-2) using weighing scale, B) Addition of catalyst CAF-2 into LSR-2, C) Placement of the original bone over the silicone mixture, D) Smearing of petroleum jelly over the lower half of silicone mold (vertebral foramen and foramina transversaria), E) Pouring of the silicone mixture to create upper half of the silicone mold, F) Separation of silicone molds, G) Inner surface of upper and lower halves of the silicone mold LSR: Liquid silicone rubber, CAF: Catalyst (Fast-acting)

It was performed for cervical vertebrae using liquid silicone rubber (LSR-2), SILOCZEST liquid silicone rubber (LSR2 FAST-500GM). It has three steps: preparation of the lower half, preparation of the upper half, and separation of molds (Figure 1).

Step 1 (Preparation of Lower Half)

A square plastic box was taken, at least 5 mm bigger than the dimensions of the bone. Petroleum jelly (release medium) was smeared inside the square plastic box. Fifty grams of LSR-2 (SILOCZEST) was taken in a plastic container (Figure 1A). Catalyst was added in the ratio of 5:1, i.e., 10 ml (Figure 1B). The mixture was thoroughly mixed unidirectionally with a wooden spoon for 5-6 seconds and poured into the square plastic box quickly (within 5-6 seconds to prevent air bubble incorporation). A wooden spoon was used to touch and remove any air bubbles formed that would rise up to the surface. The bone was gently kept on the silicone surface. Care was taken to see that the bone didn’t touch the sides as well as the base of the box. It was ensured that the silicone would reach the lower half of the foramina transversaria and vertebral foramen. The mold was allowed to harden for 15-20 minutes, till the silicone became rubbery to the touch. Once rubbery, it was considered that the process was complete (Figure 1C).

Step 2 (Preparation of Upper Half)

Petroleum jelly (release medium) was smeared over the upper surface of the prepared lower half of the silicone mold. Care was taken not to smear over the bone, to avoid adhesion of LSR to the bone, and to facilitate easy retrieval from the mold (Figure 1D). Seventy grams of LSR-2 (SILOCZEST) was taken in another plastic container. Catalyst was added in a ratio of 5:1, i.e., 14 ml. The mixture was thoroughly mixed unidirectionally, as in Step 1, and poured quickly (within 5-6 seconds to prevent air bubble incorporation) over the bone and lower half of the silicone mold in the square plastic box (Figure 1E). The upper half of the mold was allowed to harden for 15-20 minutes, till the silicone became rubbery to the touch. Once rubbery, it was considered that the process was complete.

Step 3 (Separation of Molds)

A wooden spoon was used to slide and separate the mold from the sides of the box. The plastic box was inverted and gently tapped on the table a few times to let the mold out. The two halves of the mold were gently separated using gloved fingers along the line of cleavage (Figures 1F-1G).

Bone preparation

**Figure 2:**
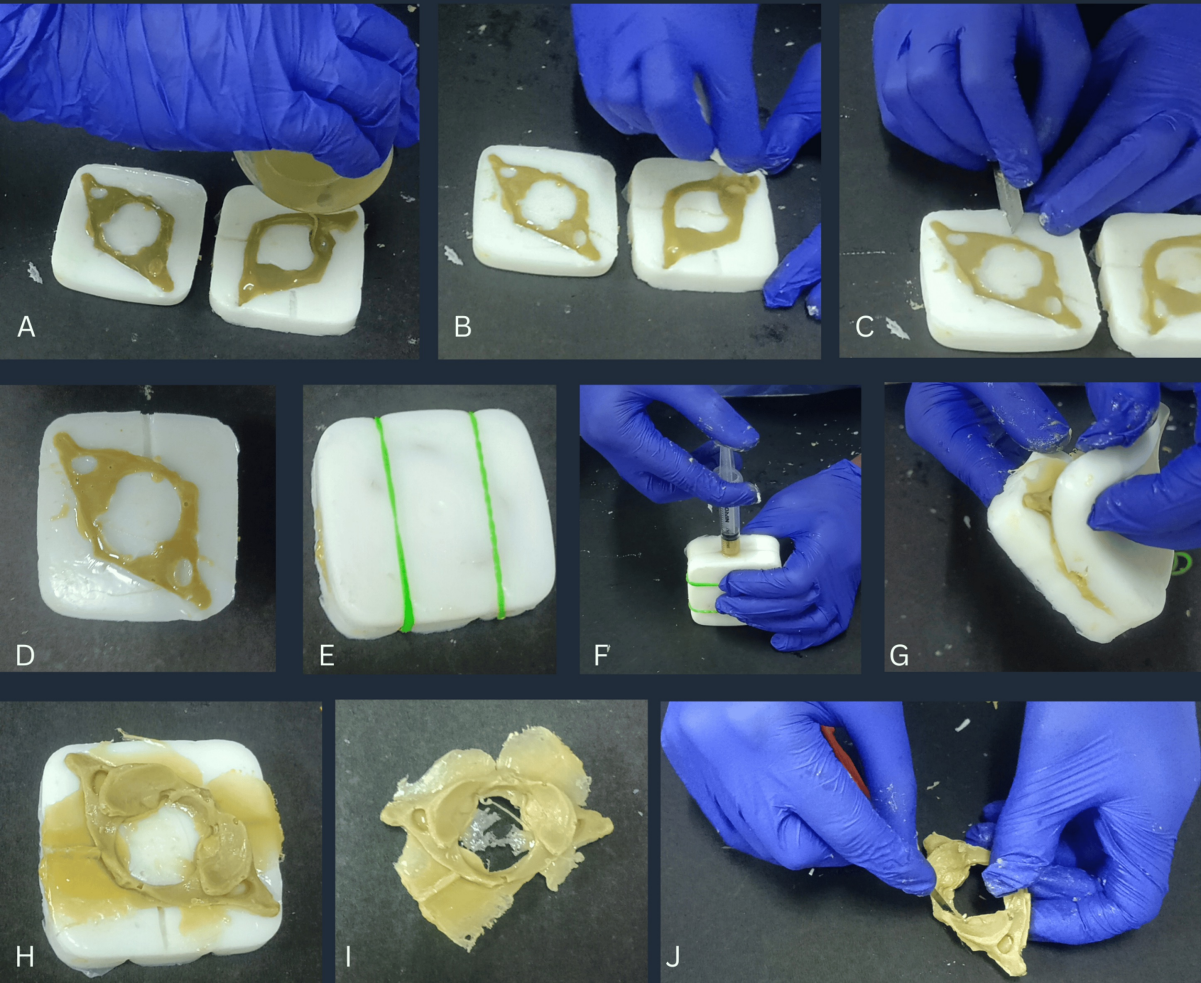
Preparation of Resin Bone A) Preparation of upper and lower halves of bone using GP resin, B) Clearing of extra resin using tissue paper, C,D) Tunnel creation in one half of the silicone mold, E) Opposition of both the halves of silicone mold using rubber bands, F) Adhesion of two halves using GP resin injected through a syringe, G) Separation of silicone molds, H) Bone retrieval from the silicone mold, I) Retrieved bone, J) Trimming of the bone GP Resin: General Purpose Resin

It was done using GP resin, which is a locally manufactured polyester or fiberglass resin meant for industrial usage. Bone preparation has four steps: preparation of the upper and lower halves, tunnel creation and opposition, adhesion of the two halves, bone retrieval, and trimming (Figure 2).

Step 4 (Preparation of Upper and Lower Halves)

To 15 g of GP resin, a touch of oil paint with semi-matte finish (Asian Paints Ltd., Mumbai, India) to match the color of the bone was added and mixed. Twenty-one drops of cobalt accelerator (locally manufactured) followed by 21 drops of Methyl Ethyl Ketone Peroxide (MEKP) catalyst (locally manufactured) were added and mixed thoroughly for 5-6 seconds. The resin mixture was poured quickly into the mold (Figure 2A). The extra resin was wiped off using tissue paper (Figure 2B). Around 5-15 minutes was given for hardening.

Step 5 (Tunnel Creation and Opposition)

A tunnel of diameter 2 mm was created on one side of the lower mold (Figures 2C-2D). The molds were opposed correctly using markings on both halves. They were kept in place using two rubber bands (Figure 2E).

Step 6 (Adhesion of Two Halves)

To 5 g of GP resin, a touch of oil paint with semi-matte finish (Asian Paints Ltd., Mumbai, India) was added and mixed. Seven drops of cobalt accelerator followed by seven drops of MEKP catalyst were added and mixed thoroughly for 5-6 seconds. The resin mixture was injected using a syringe without a needle, quickly through the tunnel (Figure 2F). The resin was given 5-10 minutes for hardening.

Step 7 (Bone Retrieval and Trimming)

Rubber bands were removed, the molds were gently separated (Figure 2G), and the bone was retrieved (Figures 2H-2I). A cutting blade was used to remove the extra resin and trim the edges of the bone (Figure 2J). Toothpicks were used to trim the edges of the foramina. Finally, the bone is ready to use.

Table 1 summarizes the quantities and curing times of materials used for artificial bone preparation.

**Table 1:** Protocol for artificial bone preparation followed in the current study LSR-2: Liquid silicone rubber-2; CAF: Catalyst (Fast-acting); GP Resin: General Purpose Resin, MEKP Catalyst: Methyl Ethyl Ketone Peroxide Catalyst The timings mentioned correspond to freshly or recently manufactured resin. Over 6 months’ time, the resin turned more viscous, requiring half the amount of accelerator and catalyst. The curing time refers to initial cure / tack free cure, adequate enough to perform all the steps. The full cure could not be ascertained due to lack of a durometer device to test the hardness of mold and bone.

S. No.	Step	Material quantity			Curing time
I.	Mold preparation	LSR-2	CAF-2		30-45 min
1.	Preparation of lower half	50 gm	10 ml		15-20 min
2.	Preparation of upper half	70 gm	14 ml		15-20 min
3.	Separation of molds	-	-		-
II.	Bone preparation	GP Resin	Accelerator	MEKP Catalyst	10-25 min
1.	Preparation of upper and lower halves	15 gm	21 drops	21 drops	5-15 min
2.	Tunnel creation and opposition	-	-	-	-
3.	Adhesion of two halves	5 gm	7 drops	7 drops	5-10 min
4.	Bone retrieval and trimming	-	-	-	-

Characteristics of artificial bone

**Figure 3:**
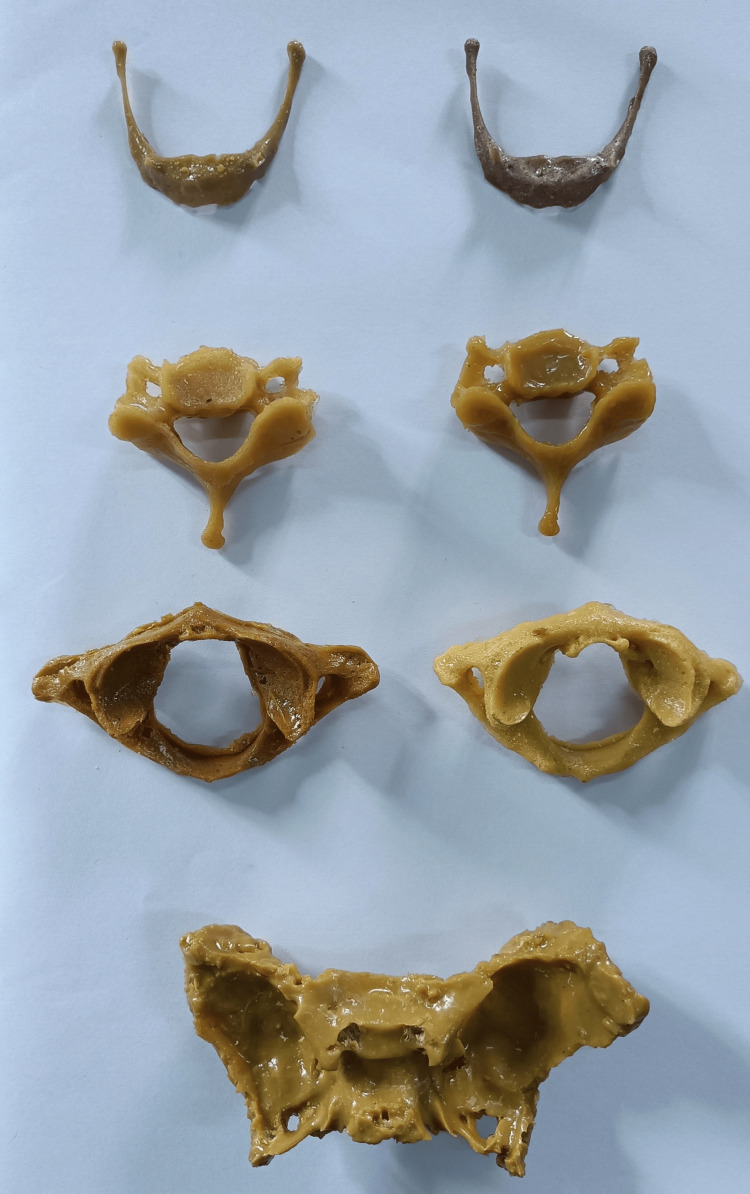
Artificial bones created using the current methodology Hyoid, typical cervical vertebra, atlas, and sphenoid bones, demonstrating all the anatomical impressions.

Bones found in scarcity, such as the hyoid, atlas, axis, typical cervical vertebra, and sphenoid bone, were created artificially (Figure 3).

As per the observations of the faculty, the artificially created bones appeared very close to the natural bone appearance. The color resembled that of the naturally procured bones. The texture appeared rugged in touch, akin to the natural bone feel. They were hard and rigid like a natural bone, and not flexible or rubbery. They do not have a sticky/slippery feel. The weight was similar to the natural bones. They were quite sturdy, withstanding force or a fall to the ground. A few breakages were observed, which could be rectified using extra resin, which could act as an adhesive, placed between the broken parts. They appeared as exact replicas of the natural bones with all finer details (e.g., the articular facets of the vertebra, the foramina transversaria, etc.). The entire procedure could be rapidly completed within 90 minutes. They were durable over time during our observation for the last year. There was no discoloration or decay. They could be easily stored, similar to natural bones. There were no safety issues; they could be handled safely using bare hands. The silicone molds were reusable for multiple artificial bone preparations.

## Discussion

Anatomy is the fundamental subject for obtaining competency in the medical profession. However, there were observed reports on anatomy teaching and student learning in crisis. This was attributed to less time given for the subject and decreased hours of cadaver dissection [6]. Almost all faculty agreed that the bone scarcity affected the learning outcome of first-year medical students [2]. The number of students handling bones to study anatomy seems to be dwindling over the years. There has been an 82% increase in the number of medical colleges and a 112% increase in MBBS seats in India between 2014 and 2023 [1].

Purchase of original bone sets involves cost, scarcity, and unethical procurement issues. This brings in the necessity of procuring artificial bones for teaching and learning osteology. These artificial bones also need to show all the anatomical structural details. With the increasing need for bone sets, and to make it economically feasible, there arises the need to probably make bones within the department premises [2].

In history, different materials were utilized to prepare anatomical models, such as wax, wood, ivory, silk, and papier-papier-mâché [7]. Various types of resins were studied to prepare artificial bones for education and surgical simulation [3,8]. The methodology using silicone and GP resin required 24 hours for the preparation of each half of the mold [3]. Our methodology described above could achieve hardening of each half of the silicone mold in about 15-20 minutes, and the resin bone hardening in 5-15 minutes. Thereby, the entire procedure could be finished within 90 minutes from the start.

The silicone molds, once prepared, can be reused many times. The locally manufactured GP resin is inexpensive. Therefore, this procedure is cost-effective. Even beginners will be able to follow the steps easily and achieve a reasonable outcome within a very short period of time. The artificial bones prepared as per our methodology were very similar to the natural bones, incorporating all the anatomical impressions. They were comparatively far better than the artificial bones procured commercially. These artificial bones require the least maintenance compared to natural bones.

The timings mentioned correspond to freshly or recently manufactured resin. Over 6 months’ time, the resin turned more viscous, requiring half the amount of accelerator and catalyst. The older resin had less working time (time to mold the resin to the desired shape) and was prone to bone breakage during manipulation. However, such broken parts could always be repaired using a small amount of resin (accelerator and catalyst added), which acted as an adhesive and bonded them together.

Long bones would require more raw materials. The irregular bones with multiple foramina, such as the base of the skull, would be difficult to prepare with this methodology. These are the limitations of our study.

Precautions to be taken

GP resin produces styrene fumes, which could cause headache, nausea, and carcinogenic effects over long-term exposure. Therefore, the resin container has to be opened in an open space or under a fume hood. Mask and gloves are advised. Direct contact of MEKP catalyst onto skin or eyes can cause corrosive burns or potential blindness [9]. Hence, nitrile gloves, gowns, and eye shields are mandatory. MEKP catalyst and accelerator should never be placed together since they can chemically react to cause an explosion [10].

## Conclusions

LSR-2 and GP Resin were used to create molds and artificial bones. The entire procedure could be completed within 90 minutes from the start, making it a rapid procedure for artificial bone preparation. This methodology is very helpful in conducting workshops to train the faculty and technicians, where the entire procedure could be finished in 90 minutes, and the outcome could be seen instantly. The current methodology is a feasible method of producing artificial bones, equivalent to natural bones. Hence, it is useful for enhancing the bone banks of respective medical colleges, tackling the bone scarcity, thereby improving the educational standards.
